# Changes in cardiac heparan sulfate proteoglycan expression and streptozotocin-induced diastolic dysfunction in rats

**DOI:** 10.1186/1475-2840-10-35

**Published:** 2011-04-25

**Authors:** Célia MC Strunz, Monique Matsuda, Vera MC Salemi, Adriana Nogueira, Antonio P Mansur, Ismar N Cestari, Monica V Marquezini

**Affiliations:** 1 Heart Institute (InCor), University of São Paulo Medical School, São Paulo 05403-000, Brazil; 2 Laboratory of Investigation in Ophthalmology, University of São Paulo Medical School, São Paulo 01246-903, Brazil; 3 Pro-Sangue Foundation and Laboratory of Experimental Air Pollution of Pathology Department, University of São Paulo Medical School, São Paulo 01246-903, Brazil

**Keywords:** Glypican, Syndecan-4, Diabetes, Cardiac Muscle

## Abstract

**Background:**

Changes in the proteoglycans glypican and syndecan-4 have been reported in several pathological conditions, but little is known about their expression in the heart during diabetes. The aim of this study was to investigate in vivo heart function changes and alterations in mRNA expression and protein levels of glypican-1 and syndecan-4 in cardiac and skeletal muscles during streptozotocin (STZ)-induced diabetes.

**Methods:**

Diabetes was induced in male Wistar rats by STZ administration. The rats were assigned to one of the following groups: control (sham injection), after 24 hours, 10 days, or 30 days of STZ administration. Echocardiography was performed in the control and STZ 10-day groups. Western and Northern blots were used to quantify protein and mRNA levels in all groups. Immunohistochemistry was performed in the control and 30-day groups to correlate the observed mRNA changes to the protein expression.

**Results:**

In vivo cardiac functional analysis performed using echocardiography in the 10-day group showed diastolic dysfunction with alterations in the peak velocity of early (E) diastolic filling and isovolumic relaxation time (IVRT) indices. These functional alterations observed in the STZ 10-day group correlated with the concomitant increase in syndecan-4 and glypican-1 protein expression. Cardiac glypican-1 mRNA and skeletal syndecan-4 mRNA and protein levels increased in the STZ 30-day group. On the other hand, the amount of glypican in skeletal muscle was lower than that in the control group. The same results were obtained from immunohistochemistry analysis.

**Conclusion:**

Our data suggest that membrane proteoglycans participate in the sequence of events triggered by diabetes and inflicted on cardiac and skeletal muscles.

## Background

Diabetes mellitus is a complex disorder resulting in large- and small-vessel disease and impaired organ function. It is characterized by hyperglycemia and by a variety of end-organ damage [1].

One of the major causes of morbidity and mortality in diabetic patients is the cardiovascular disease related to the myocardial contractile system [2], with diastolic dysfunction being an early event of diabetic cardiomyopathy, preceded by a change in cardiac metabolism [3,4].

Diabetic cardiomyopathy remains a poorly understood disease. Its development results in myocardial fibrosis and collagen deposition, which may lead to altered myocardial relaxation and diastolic dysfunction [5]. Several mechanisms were proposed to explain the installation of the disease, but the main changes are promoted by hyperglycemia, and represent an adaptive or maladaptive response that culminates in the installation of this clinical entity [5].

Furthermore, endothelial dysfunction may contribute to the development of cardiopathy through the under- or overproduction of growth factors [6].

In animal models of Type 1 diabetes induced by streptozotocin (STZ), the cardiac contractile dysfunction appears to be related to prolonged hypoinsulinemia and hyperglycemia [7].

Heparan sulfate proteoglycans (HSPGs) are highly charged macromolecules found on the surface of virtually every cell type. The main cell surface proteoglycans carrying heparan sulfate in mammalian cells are syndecans and glypicans. They interact with a wide variety of molecules, including extracellular matrix components (ECM), enzymes, and growth factors, participating as regulators of biological processes, ranging from embryogenesis to hemostasis [8,9]. As a consequence, they are involved in different pathological conditions, such as wound repair, cancer, atherosclerosis, and thromboembolic disorders [8-10]. Moreover, studies in diabetes have correlated nephropathy and retinopathy microvascular disease with lower amounts of HSPGs in the endothelial basement membrane [11-14]. A reduction in heparan sulfate (HS) and proteoglycan synthesis in cultured epithelial cells in the presence of high-glucose medium has been shown [15].

However, a gap exists in our understanding of HSPG alterations in other tissues during diabetes. Considering the changes in some HSPGs' expression in renal and vascular tissues, we hypothesized that similar changes may happen in the skeletal and cardiac muscles.

The aim of this study was to investigate whether alterations in myocardial function during hyperglycemia by insulin depletion are accompanied by changes in the expression of HSPGs in cardiac muscle and, if so, whether the change is muscle specific or a general response to the disease. For this purpose, we examined the mRNA expression and the amount of 2 key cell surface heparan sulfate chain-carrying core proteins, syndecan-4 and glypican-1, after 24 hours, 10 days, and 30 days of STZ-induced experimental diabetes in 2 muscles, cardiac and skeletal. HSPGs expression was evaluated in skeletal muscle, because it is the major organ responsible for glucose uptake under insulin-stimulated conditions and it is affected by the metabolic deregulation observed in diabetes. As happens in cardiac muscle, the contractile dysfunction could be detected in skeletal muscle caused by degeneration and necrosis of myofibers together with type II atrophy [16,17].

## Methods

### Animals

This work was performed in compliance with the ARRIVE guidelines on animal research [18]. All experimental procedures were performed in accordance with the Guidelines for Ethical Care and Use of Experimental Animals and approved by the institution's ethics committee. Diabetes was induced in overnight-fasted rats by a single intravenous injection of 50 mg/kg of STZ in citrate buffer (Sigma Chemical Co., St. Louis, MO, USA). Diabetes induction by STZ injection is a well-characterized model of experimental Type 1 diabetes, where the selective destruction of beta-cells of pancreatic islets promotes a permanent hyperglycemic state and consequently diabetes complication [19]. After STZ injection, all animals were returned to their cages and kept in a ventilated shelf under a controlled 12-hour light/dark cycle and temperature (21-24°C).

Sixteen male Wistar rats obtained from the central animal facility of the University of São Paulo Medical School weighing 200-300 g were randomly divided into 4 groups of 4 animals each according to time of diabetes induction: control (sham injection with saline), 24 hours, 10 days, and 30 days after STZ injection.

All animals were sacrificed with a lethal dose of sodium pentobarbital (60 mg/kg i.p.) and had the heart and the gastrocnemius muscles immediately removed, washed in ice-cold 0.9% NaCl solution, weighed, frozen in liquid nitrogen, and stored at -80°C.

### Echocardiography

High-resolution echocardiography was performed in 4 diabetic rats after 10 days of induction and in 4 rats of the same age and sex from the control group just before sacrifice. Transthoracic echocardiography was performed after anesthesia with pentobarbital (40 mg/kg; i.p.) using a Sequoia 512 machine (Acuson, Mountain View, CA) equipped with a 13-MHz linear-array transducer. Images were stored digitally on magneto-optical discs [20].

Left ventricular end-systolic (LVESD) and end-diastolic dimension (LVEDD), interventricular septal thickness (IVST), and posterior wall thickness (PWT), both in diastole, were measured at the level of the papillary muscles on the short-axis view using 2-D guided M-mode imaging [20,21]. All measurements were obtained according to American Society of Echocardiography recommendations [22]. Three representative cardiac cycles were analyzed and averaged for each measurement. Fractional shortening was calculated from the M-mode recordings, as previously described [23]. Left ventricle (LV) mass was calculated from M-mode recordings by using the uncorrected cube formula, assuming spherical LV geometry [20]. The LV mass index was determined as the ratio of LV mass in grams to body weight in grams.

The peak velocity of early (E) and late (A) diastolic filling and E/A ratio, deceleration time of the E wave (DT), and isovolumic relaxation time (IVRT) were obtained from the mitral inflow recordings, as previously described [20]. DT and IVRT were obtained in animals when the E and A waves were not fused. We also measured systolic (S'), early (E'), and late (A') diastolic peak velocities of Doppler tissue imaging with a sample volume placed at the septal side of the mitral annulus in the apical 4-chamber view.

### Western blot

Proteoglycans (PGs) were extracted from the cardiac or skeletal muscle pool obtained from all 4 animals of each experimental group according to the protocol described elsewhere [24]. After extraction, the supernatants were dialyzed and purified in DEAE Sephacel (Sigma) and protein concentration determined using Bradford's method [25]. Samples underwent digestion with a mixture of 0.06 unit of chondroitinase ABC and 1 unit of heparitinases (Sigma) at 37°C for 4 hours to remove glycosaminoglycans from the protein core [26].

Ten μg of PGs underwent 7.5% SDS-PAGE, followed by electrotransfer to an Immobilon-P membrane (Millipore, Belford, MA, USA). Membranes were blocked and incubated with the primary antibody, either antiglypican-1 C-18 or antisyndecan-4 N-19 (1:1000; Santa Cruz Biotechnology, Inc., Santa Cruz, CA, USA), followed by the secondary antibody, goat anti-rat IgG-HRP (1:5000; Santa Cruz Biotechnology, Inc). The blots were developed using a chemiluminescent reagent (Western Blotting Chemiluminescence Luminol reagent; Santa Cruz Biotechnology) and exposed to Kodak MGX/Plus film (Eastman Kodak, Rochester, NY, USA). The PGs were quantified with the Eagle Eye analyzer system (Stratagene, La Jolla, CA, USA). Mean and SE were calculated using 4 independent experiments. The results were expressed as arbitrary optical density units (OD).

### RNA isolation from cardiac and skeletal muscles

Total RNA was isolated from the heart apical region and skeletal muscle by using TRIzol Reagent (Invitrogen, Carlsbad, CA, USA) as described in the manufacturer's protocol. RNA integrity was determined by agarose gel electrophoresis [27].

### RT-PCR assays

cDNA was synthesized using total RNA from cardiac and skeletal muscles by the Superscript Pre-amplification System (Invitrogen) and amplified by PCR using the manufacturer's protocol. PCR primers used in this study are listed in Table 1 and were designed using previously published sequences [28-30]. The samples were amplified for 35 cycles (denaturation at 94°C for 45 seconds; annealing at 58°C for 1.5 minutes, extension at 72°C for 1.5 minutes). All amplifications were carried out using a thermal cycler (MJ Research Inc. Watertown, MA, USA). β-actin was used as a control.

**Table 1:** Oligonucleotide sequences and expected PCR product sizes

Oligonucleotide sequence			Product length (bp)
Glypican [28]	sense:	5' TTG GCA GTG TGC ATA TGT G 3'	700
	anti sense:	5' GTG AAC AGG AAG AGC AGA AAG 3'	

Syndecan-4 [29]	sense:	5' CGA GAG ACT GAG GTC ATA G 3'	471
	anti sense:	5' TCG TAA CTG CCT TCA TCC 3'	

ß-actin [30]	sense:	5' ATC ATG TTT GAG ACC TTC AAC AC 3'	890
	anti sense:	5' TCT GCG CAA GTT AGG TTT TGT C 3'	

### Cloning and sequencing of PCR products

The PCR products were purified with a Concert Rapid Gel Extraction System (Invitrogen) and cloned with a pGEM-Easy cloning vector (Promega, Madison, WI, USA.). The clones were identified by blue/white selection, processed for plasmid purification with the Wizard Plus kit (Promega), and sequenced (ABI PRISM- 377 DNA Sequencer- Perkin Elmer, Foster City, CA, USA) by using DNA prepared according to the protocol of the sequencing kit DNA Big Dye Terminator (Applied Biosystem- Perkin Elmer, Norwalk, CT, USA). The identity of the products was verified by using the basic local alignment search tool (BLAST) on the GenBank database.

### Northern blot

Total tissue RNA (10 μg) from 4 animals of each group (control, 24-hour, 10-day, and 30-day) was fractionated in 1% agarose-formaldehyde gel [31]. The RNA was transferred to a positively charged membrane (Duralon-UV Stratagene) and prehybridized in a Stratagene hybridization solution and hybridized with digoxigenin (Dig)-labeled probes, syndecan-4 and glypican, prepared according to instructions of the Dig DNA labeling kit (Roche Molecular Biochemicals, Mannheim, Germany). The blots were revealed with a Dig luminescent detection kit (Roche) and exposed to Kodak MXG/Plus film. The hybridization signals were quantified with the Eagle Eye system, and normalized to the amount of 28S rRNA in the samples. Results are presented as mean ± SE of the relative density (optical density of proteoglycan/optical density of 28S rRNA * 100) of samples from 4 rats in each group. The experiment was run in duplicate.

### Immunohistochemistry

For histological analysis, 5-μm sections were obtained from formalin-fixed paraffin-embedded samples of cardiac and skeletal muscle from control rats and the 30-day group and routinely stained with hematoxylin-eosin.

Three-micrometer sections from these specimens were deparaffinized, rehydrated, and underwent antigen retrieval in 0.5% pepsin pH 1.8 for 30 minutes at 37°C. The specimens were then incubated in 3% aqueous hydrogen peroxide for 30 minutes to quench endogenous peroxidase activity. Incubation with 1% BSA and 5% fetal calf serum in Tris-HCl, pH 7.4 for 60 minutes at room temperature was performed to suppress nonspecific binding of subsequent reagents. The sections were then incubated with the primary antiglypican-1 C-18 and antisyndecan-4 N-19 (Santa Cruz Biotechnology Inc.) diluted 1:200. The Catalyzed Signal Amplification system (CSA system, HRP, Dako, Carointeria, CA, USA) was used and consisted of 15-minute sequential incubations with a biotinylated link, streptavidin complex, amplification reagent, and streptavidin peroxidase. Each of these 15-minute incubation steps was preceded by a 5-minute rinse with 1% Tween 20 Tris-HCl, pH 7.4. Staining was completed by 3-minute incubation with 3' -3 -diaminobenzidine tetrachloride (DAB; Sigma, St Louis, MO, USA), which resulted in a brown colored precipitate at the antigen sites. The specimens were then lightly counterstained with Mayer's hematoxylin, dehydrated, and xylene-based mounted under glass cover slips. Negative controls were treated as above, but a solution of 1% BSA in Tris-HCl, pH 7.4 replaced the primary antibody. Epithelium and blood vessels were considered internal positive controls.

### Statistical analysis

The results are expressed as mean ± SE. Data were compared using analysis of variance (ANOVA) followed by the Tukey Multiple Comparison Test using Graf Pad Prism for Windows software (Graft Pad Software Inc., San Diego, CA, USA). The *t *test was applied for echocardiographic results. P < 0.05 was considered statistically significant.

## Results

### Animal characteristics

All STZ-treated animals developed severe hyperglycemia (> 27.0 mmol/L), including the 24-hour group, compared with the control (nondiabetic) group (< 7.5 mmol/L). Body weight was significantly lower in 10-day and 30-day groups compared with control animals according to ANOVA followed by the Tukey Multiple Comparison Test: 217.4 ± 3.9 g and 222.4 ± 9.53 g vs 265.8 ± 10.9 g, P < 0.05.

### Echocardiographic indices show impaired relaxation

Left atrium dimension, LVESD and LVEDD, IVST, and PWT, LV fractional shortening, and LV mass index were not statistically different in the control and 10-day groups (Table 2). Although LV fractional shortening was normal, S' peak velocity was decreased in the diabetic group (Table 2). This finding shows that Doppler tissue imaging is a useful tool for the assessment of systolic function in this model of diabetic myocardial disease and is altered earlier than the standard indices of global systolic function.

**Table 2:** Echocardiographic variables for control and diabetic groups after 10 days of induction

Variables	Control group (n = 4)	Diabetic group (n = 4)	P value
Left atrium dimension (mm)	2.8 ± 0.3	3.0 ± 0.7	NS

Interventricular septum thickness (mm)	1.9 ± 0.2	1.9 ± 0.3	NS

Posterior wall thickness (mm)	1.8 ± 0.2	1.8 ± 0.4	NS

LV end-diastolic dimension (mm)	5.3 ± 0.1	5.0 ± 0.1	NS

LV end-systolic dimension (mm)	2.8 ± 0.1	3.1 ± 0.1	NS

LV fractional shortening	0.48 ± 0.1	0.37 ± 0.2	NS

LV mass index (g/body weight)	2.4 ± 0.5	2.1 ± 0.2	NS

Isovolumic relaxation time (ms)	28 ± 2	59 ± 9	< 0.001

E peak velocity (cm/s)	80 ± 10	50 ± 14	< 0.05

S' peak velocity (cm/s)	3.8 ± 0.5	2.5 ± 0.5	< 0.05

E/E'	13 ± 2	14 ± 5	NS

LV = left ventricular; S' = systolic septal mitral annulus peak velocity.

*t *test, NS = nonsignificant; P < 0.05.

Diastolic function indices allowed the differentiation between groups, because E peak velocity was decreased in the diabetic group, and IVRT was increased in the same group. No difference occurred between diabetic and normal groups for E/E' ratio (Table 2). Taken together, the diastolic function indices reflect an impaired relaxation with normal LV end-diastolic pressure.

### Membrane HSPGs' mRNA is present in normal cardiac and skeletal muscle

We detected the presence of glypican-1 and syndecan-4 mRNA in normal cardiac and skeletal muscles by RT-PCR. The identity of the HSPGs cDNA was confirmed by sequence comparison with published sequences for these molecules [GeneBank accession n° **L02896**and **M81786**].

### Glypican-1 concentration increases in cardiac muscle and decreases in skeletal muscle

**Figure 1:**
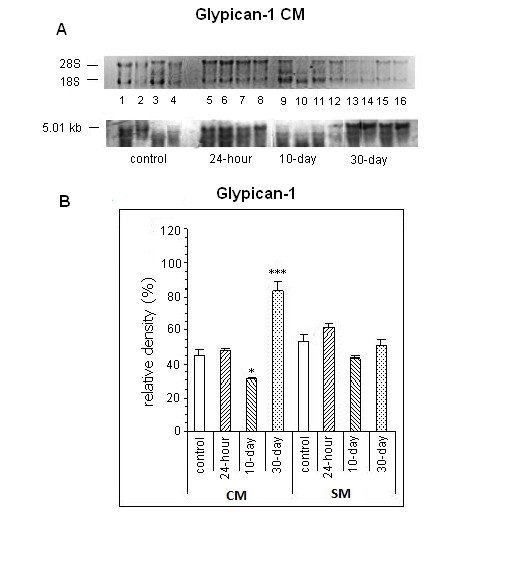
**Northern blot analysis of glypican-1 from cardiac muscle (CM) and skeletal muscle (SM)**. Membrane of glypican mRNA from CM (A) stained with ethidium bromide (upper) and hybridized with glypican labeled probe (lower). B. Bars represent the mean values ( ± SE) of relative density (optical density of proteoglycan/optical density of 28S rRNA * 100) from CM and SM in the 4 groups. *P < 0.05; ***P < 0.001 compared with control.

A single mRNA band was detected for glypican in each tissue: 5.01 kb in heart tissue (Figure 1A) and 5.36 kb in skeletal muscle (not shown). Glypican mRNA expression in cardiac muscle revealed, in comparison to normal tissue, decreased values in the 10-day group (relative density: 30.40% ± 1.08% vs 44.90% ± 4.02%; P < 0.05) followed by a significant increase, almost 100%, in the 30-day group (83.80% ± 5.07% vs 44.90% ± 4.02%; P < 0.001). STZ treatment did not change mRNA levels in skeletal muscle (Figure 1B).

Protein analysis showed the presence of 2 bands of molecular mass (Mr) of 50 and 57 kDa in cardiac and skeletal muscles. The bands obtained in our study are probably related to the presence of reduced and nonreduced glypican core proteins. Similar data were reported for glypican extracted from tumor cell lines in an immunoblot analysis that had a band of 48 kDa at nonreducing conditions and 55 kDa at reducing, quite similar to the molecular masses obtained in our study [32,33].

**Figure 2:**
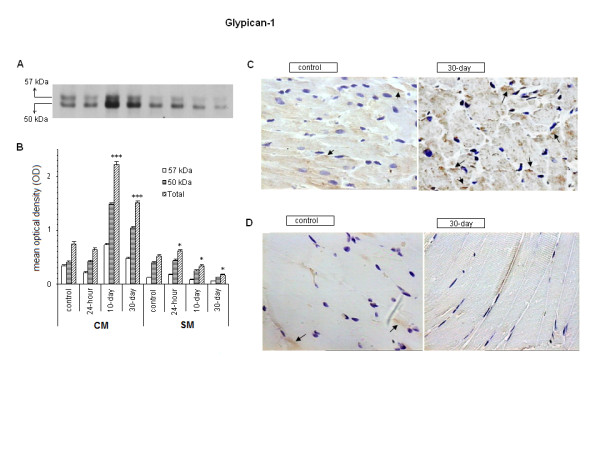
**Expression of glypican-1 in CM (cardiac muscle) and SM (skeletal muscle)**. A. Typical Western blot of 7.5% SDS-PAGE using antiglypican antibodies in extracts of muscles of control, 24-hour, 10-day, and 30-day induced rats. B. Bars represent the mean values ( ± SE) of arbitrary optical density units (OD) of 4 independent blots (*P < 0.05; ***P < 0.001 compared with control). C and D. Immunohistochemistry from CM and SM for control and 30-day specimens. Arrows indicate the brown-colored precipitates.

As for the mRNA expression, an increase was noted in the amount of glypican core protein in cardiac muscle accompanying the development of diabetes, although this elevation could be detected not only in the 30-day group but also in the 10-day group, OD: 2.219 ± 0.06 and 1.520 ± 0.031, respectively, vs OD: 0.742 ± 0.0422; P < 0.001 (Figure 2A and 2B). On the other hand, our results obtained from skeletal muscle showed a small increase in the 24 hour-group (OD: 0.618 ± 0.023 vs 0.517 ± 0.030; P < 0.05) followed by a decrease of this PG in the 10- and 30-day groups (OD: 0.333 ± 0.020 and 0.178 ± 0.011), respectively, compared with the control, OD: 0.517 ± 0.030; P < 0.05 (Figure 2A and 2B). The immunohistochemistry qualitative analysis confirmed the higher level of glypican in cardiac muscle of the 30-day group compared with the control group (Figure 2C). For skeletal muscle, as expected, the analysis of the sections showed the presence of a few spots of colored precipitate in the control and the absence of them in the 30-day group, likely because of the lack of sensitivity of this method for such a low amount of PG (Figure 2D).

### Syndecan-4 amounts increase in cardiac and skeletal muscles during experimental diabetes progression

**Figure 3:**
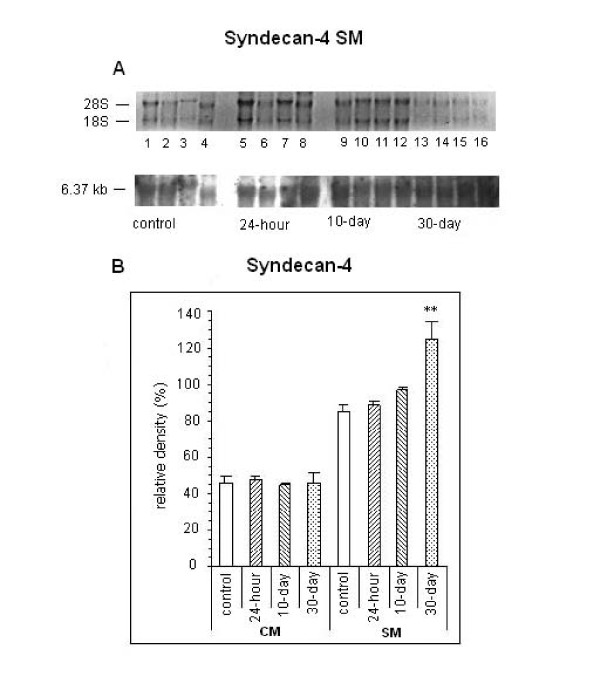
**Northern blot analysis of syndecan-4 from cardiac muscle (CM) and skeletal muscle (SM).** Membrane of syndecan mRNA from CM   (A) stained with ethidium bromide (upper) and hybridized with syndecan labeled probe (lower). B. Bars represent the mean values ( ± SE) of relative density (optical density of proteoglycan/optical density of 28S rRNA * 100) from CM and SM in the 4 groups. **P < 0.01 compared with control.

RNA blot filters hybridized with a rat syndecan-4 specific DNA probe revealed the presence of 2 bands (6.57 and 3.34 kb, not shown) for adult cardiac and one band for skeletal muscle, 6.37 kb (Figure 3A). Quantification of syndecan-4 mRNA expression in skeletal muscle revealed an increase of almost 50% in the 30-day group (relative density: 125.00% ± 10.60% vs 84.80% ± 5.98%; P < 0.01) compared to controls. On the other hand, for cardiac tissue we did not detect any changes between the samples tested (Figure 3B).

**Figure 4:**
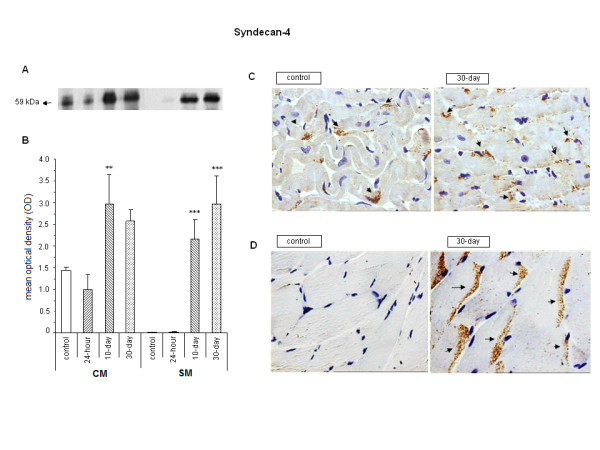
**Expression of syndecan-4 in CM (cardiac muscle) and SM (skeletal muscle)**. A. Typical Western blot of 7.5% SDS-PAGE using antisyndecan-4 antibodies in muscles of control, 24-hour, 10-day, and 30-day induced rats. B. Bars represent the mean values (± SE) of arbitrary optical density units (OD) of 4 independent blots (**P < 0.01; ***P < 0.001 compared with control). C and D. Immunohistochemistry from CM and SM for control and 30-day specimens. Arrows indicate the brown-colored precipitates.

The protein analysis revealed a band of Mr 59 kDa, with a similar pattern for cardiac and skeletal muscles (Figure 4A and 4B). This molecular mass may represent a homo- or heterooligomerization of the protein core, quite similar to that observed by Hamon et al. (Mr 56 kDa) [34]. Although the mean optical density value obtained for syndecan from heart muscle in the 30-day group almost doubled compared to that in control, it was not statistically significant, probably because of the high variability of the results in this group. On the other hand, for the 10-day group the increase was significant (OD: 2.971 ± 0.666 vs 1.435 ± 0.072; P < 0.01). The same pattern was detected for skeletal muscle with an increase in protein level in the 10- and 30-day groups, OD: 2.160 ± 0.433 and 2.845 ± 0.643, respectively, compared with the control group, OD: 0.011 ± 0.005, P < 0.001. It must be emphasized that, in skeletal samples, the amount of this PG was low not only in the control but also in the 24-hour group (Figure 4A and 4B). The qualitative analysis of immunohistochemistry (control and 30 days after induction) confirmed the increase observed in the Western blot for the 2 muscles analyzed (Figure 4C and 4D).

### ß-actin expression is not altered in cardiac and skeletal muscles

HSPG mRNAs changes induced by STZ treatment were not paralleled by alterations in ß-actin mRNA from heart and skeletal muscle (not shown).

## Discussion

In the present study, the echocardiography obtained from diabetic male rats after 10 days of induction pointed to signs of diastolic dysfunction with delayed LV relaxation but normal LV end-diastolic pressure. The same results were observed by Braga et al. [35] for chronic type 2 diabetes mellitus normotensive patients who had higher values of IVRT and lower E values compared with nondiabetic patients.

Other studies of early diabetic cardiomyopathy using experimental models of diabetes demonstrated echocardiographic systolic and diastolic functional impairment in a group of 14-week-old Zucker diabetic fatty rats (ZDF) [36,37]. As in our case, prolonged IVRT and decreased E peak velocity indicating an impaired LV relaxation were observed by them [36]. They established the relation between changes in right ventricle (RV) and LV insulin-stimulated glucose utilization and systolic dysfunction suggesting that diabetes affects both ventricles [37].

We did not observe the presence of global systolic dysfunction, probably due to the experimental diabetic rat model we used, type 1 diabetes (SZT-induced rats) versus type 2 (ZDF). Also, as an effort to demonstrate the earlier heart changes, our model of acute diabetes induction led us to perform the echocardiographic tests in rats after only 10 days of induction, which probably is too early to find the alterations registered for the 14-week-old chronically diabetic rats.

On the other hand, considering the review by Pulinilkunnil & Rodrigues [38] and the study of Zhong et al. [39], for whom the evidence of cardiac malfunction was detected after 4-6 weeks, the impairment in cardiac function observed by us after 10 days could be considered too precocious. Besides the methodological differences between the studies, we must take into account that this dysfunction could be caused by the direct action of STZ towards the myocytes as suggested elsewhere [40].

Our data confirm the presence of syndecan-4 and glypican-1 mRNAs in cardiac and skeletal muscle in control rats, which is in agreement with other findings in the literature [41-45]. Concerning the protein core, syndecan-4 is abundant in cardiac muscle but could barely be detected in skeletal muscle from control animals under our experimental conditions as shown by Western blot and immunohistochemistry analysis, differently from its mRNA expression.

In our study, after diabetes induction, an increase occurred in syndecan-4 mRNA and protein expression in skeletal muscle. The initial low abundance of this PG in normal adult tissue is in agreement with reports in the literature [44] that describe its presence only in skeletal satellite cells. The observed increase in syndecan-4 expression after induction may be related to muscle breakdown and repair.

Temporal and spatial increases in the expression of syndecans during wound-healing processes, including skin and arterial injuries, and myocardial infarction have been demonstrated [8,46]. The biological activity of HSPGs is thought to be largely due to the presence of attached heparan sulfate chains capable of binding growth factors, like basic fibroblast growth factor (b-FGF) [8]. In hyperglycemia, the b-FGF glycation is enhanced, which reduces its mitogenic activity and may explain the impaired wound healing, angiogenesis, and microangiopathies that occur in diabetes [47,48]. Data from the literature show that HSPG-bound b-FGF is resistant to nonenzymic glycation-induced loss of activity [15]. So, the increased expression of HSPGs in diabetes would protect b-FGF from inactivation, which would confer on these molecules a regulatory role in the control of biological processes in which FGFs are involved [49].

b-FGF that is localized in the cytoplasm of myofibers is accurately targeted, both in time and space, to sites of mechanical injuries [50,51], which could explain alterations in expression and/or the amount of a particular HSPG regarding its spatial distribution.

No difference was found between control and diabetic rats in glypican-1 mRNA extracted from skeletal muscle. On the other hand, the data obtained from protein analysis pointed to the presence of lower levels of glypican-1 in diabetic rats compared with controls. Lower amounts of this membrane-bound proteoglycan, as happens to other HSPGs from extracellular matrix, could promote a change in charge barrier densities, because of the loss of anionic sites [11-14] and may be involved in plasma membrane wounding.

Glypican and syndecan-4 are both present on the surface of myotubes [38,41,52], so if the variation observed for some HSPGs obeys a spatial model, we would expect the same pattern for these 2 PGs, which did not happen. Liu et al. [53] showed that glypican and syndecans could have distinct functions, even when expressed by the same cell type. Different functions could explain our results, considering that syndecan-4 appears to be a requirement for a functional response for proliferation that may be mediated by FGFs, being coexpressed with this growth factor in satellite cells [44].

So, probably, different patterns of expression triggered by the development of diabetes with a lower level for glypican and a higher level for syndecan, would reflect distinct functions.

In cardiac muscle from normal rats, the presence of glypican-1 and syndecan-4 detected in our study is in agreement with reports in the literature, which localized them in cardiomyocyte plasma membranes [43,45].

The increased level of syndecan-4 specific protein after diabetes induction was not paralleled by the mRNA expression in cardiac cells. The same happened for glypican-1, whose pattern for mRNA, with a relative density in the 10-day group lower than that in the control, was not followed by protein analysis, which had raised levels after STZ-induction. One possible mechanism for these conflicting results could be a decrease in the rate of degradation of these HSPGs or the stabilization of their transcripts. Syndecan expression can also be controlled posttranscriptionally as happens to syndecan-3, the transcript of which is abundant in rat heart tissue, whereas the protein molecule is hardly detectable [54].

Data in the literature establish the relationship between increased levels of b-FGF, cardiac hypertrophy, and reversion of muscle structural mRNA to the fetal isoforms, conferring to FGF a crucial role in the cardiac hypertrophy process [55,56].

The increased amount in diabetes of glypican-1 and syndecan-4 is somewhat expected, because these proteoglycans modulate the interaction of FGF, augmented in diabetes, with different receptors and protect it against inactivation, as explained earlier [57]. Moreover, the presence of glypican-1 expression corresponds in most cases to locations of high mitotic activity, where HS-dependent and heparin binding growth factors are known to play important developmental roles [58].

Concerning syndecan-4, it is present in the costamere, a unique cytoskeletal adhesion complex present in striated muscle, and in focal adhesion in adult cardiomyocytes [45]. Likewise, b-FGF receptor was localized in costameres, bestowing on syndecan 4 a possible role as a regulator of b-FGF co-receptor interactions with cardiomyocytes [59]. In addition, it has been suggested that this proteoglycan participates in the transmission of contractile force to the collagenous extracellular matrix [45]. As discussed earlier, contractile dysfunction is an important event in diabetic cardiomyopathy, so alterations in molecules involved in the transmission of myocyte contraction to ECM might be expected.

In our investigation, we did not include female rats, because of the potential cardiovascular protective influence of estrogen. Recently, it was shown that in ovariectomized mRen2. Lewis female rats, an after menopause model, the estrogen depletion promotes the worsening of the diastolic dysfunction with impaired LV relaxation and an increase of myocardial collagen deposition [60].

The small number of animals investigated must be registered as a limitation of this study.

## Conclusion

The results obtained for glypican in cardiac and skeletal muscle samples pointed to a muscle-specific response of this proteoglycan related to development of diabetes. Our data did not allow us to determine a causative relationship between the alterations observed in both proteoglycan expression and the in vivo functional changes observed. However, it is clear that the diastolic dysfunction, an early sign of cardiac involvement, and the change in these cell surface sulfated proteins occur, at least, as parallel events.

Because the processes involved in the disease are not mutually exclusive, the identification of any event linked to the development of diabetic cardiomyopathy will help the understanding of the pathophysiology of the disease and may contribute to a future clinical approach.

This is the context of this study: to help in the identification of molecules that with a reasonable amount of certainty participate in the sequence of events that culminate in the myocardial insult inflicted by diabetes. So determining the trigger of this clinical entity should be a goal for researchers, because it would lead us to the possibility of intervening through specific therapies.

## List of abbreviations

b-FGF: basic fibroblast growth factor; DT: deceleration time of the E wave; HSPG: heparan sulfate proteoglycan; IVRT: isovolumic relaxation time; IVST: interventricular septal thickness; LVEDD: end-diastolic dimension; LVESD: left ventricular end-systolic; PWT: posterior wall thickness; STZ: streptozotocin; ZDF: Zucker diabetic fatty rats.

## Competing interests

The authors declare that they have no competing interests.

## Authors' contributions

CMCS participated in the study design, performed the experiments, analysed data, interpreted results and drafted the manuscript. MM and AN participated in various experiments. VS carried out the echocardiographic experiments. APM critically reviewed the manuscript. INC participated in the study design, animals experiments, molecular biology analysis, and critically reviewed the manuscript. MVM conceived the study design, performed the experiments, and participated in data analysis and interpretation.

All authors read and approved the final manuscript.
